# Are Women in Turkey Both Risks and Resources in Disaster Management?

**DOI:** 10.3390/ijerph120605758

**Published:** 2015-05-26

**Authors:** Özden Işık, Naşide Özer, Nurdan Sayın, Afet Mishal, Oğuz Gündoğdu, Ferhat Özçep

**Affiliations:** 1 Istanbul Neighborhood Disaster Volunteers (MAG), Istanbul 34524, Turkey; E-Mail: ozden2010@gmail.com; 2 AFAD (Emergency and Disaster Management Department), Istanbul Governorship, Istanbul 34410, Turkey; 3 Division of Seismology, Department of Geophysics, Istanbul University, Istanbul 34320, Turkey; 4 Division of Earth Physics, Department of Geophysics, Istanbul University, Istanbul 34320, Turkey; E-Mails: nursayin@istanbul.edu.tr (N.S.); gundogdu@istanbul.edu.tr (O.G.); ferozcep@istanbul.edu.tr (F.O.); 5 Department of Health, Istanbul University, Istanbul 34740, Turkey; E-Mail: afetmishal@hotmail.com

**Keywords:** gender inequality, gender sensitivity, gender barrier, disaster management, risks, resources, cultural feminism, universal culture of disaster

## Abstract

From a global perspective, the universality of gender-related societal issues is particularly significant. Although gender inequality is considered a sociological problem, the large number of female victims in disasters warrants an assessment of disaster management sciences. In this article, related concepts are discussed based on their relevance sociologically and in disaster management to develop a common terminology and examine this complex topic, which is rooted in different social profiles and anthropological heterogeneity throughout the world. A brief history is discussed, and significant examples are provided from different disasters in Turkey to illustrate why a woman-oriented approach should be adopted when evaluating concepts of gender inequality. Observations of disasters have shown that it is important to apply international standards (humanitarian charter and minimum disaster response standards), especially during periods of response and rehabilitation. Relevant factors related to gender should be included in these standards, such as women’s health and hygiene, which will be discussed in more detail. A woman-based approach is designed in relation to two aspects: risks and resources. Thus, gender-sensitive methods of mitigating and preventing disasters are provided. The main purpose of the article is to contribute to the development of a universal culture that prioritizes gender in disaster management.

## 1. Introduction

Within a legal framework, a disaster is defined by Gündoğdu and Özçep [1] as “a natural or human-driven event that interrupts the normal life and social activities of humans along with other living beings, causing physical, socio-cultural and economical losses, and cannot be overcome by the affected society.” More briefly, a disaster is a lack of resources in response to an event. These resources include teams, equipment and humans; of these resources, humans are the most valuable. Therefore, ensuring the availability of the necessary human resources is the key element in reducing losses in disasters [2].

Successful disaster management is only possible by foreseeing risks and optimizing the use of resources [2]. Ergünay [3] defines contemporary disaster management as “a fight that requires the recognition of potential dangers and risks associated with disasters and rational precautionary methods to prevent or minimize the damages from such dangers and risks prior to a disastrous event.” Ergünay also states that every person, from citizens to the highest authorities, has a duty and responsibility in this regard. However, site observations in regions that have been affected by disasters reveal that women and men are not affected equally by disasters due to unequal opportunities in disaster management education based on gender stratification in male-dominant societies. Furthermore, in such societies, women have additional dependencies and household responsibilities (e.g., child care, feeding) that arise from their roles as mothers. Thus, women face greater risks in disasters compared to men. However, women may also be more important resources than men in post-disaster recovery and rehabilitation [4], indicating that a gender-based perspective in disaster management is necessary.

**Figure 1:**
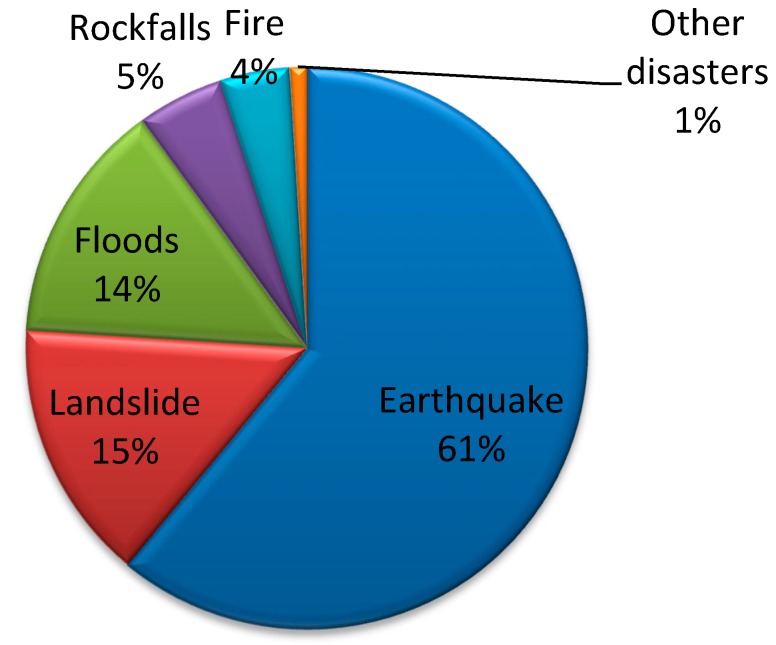
A comparison of different types of disasters in Turkey (after [5]).

**Figure 2:**
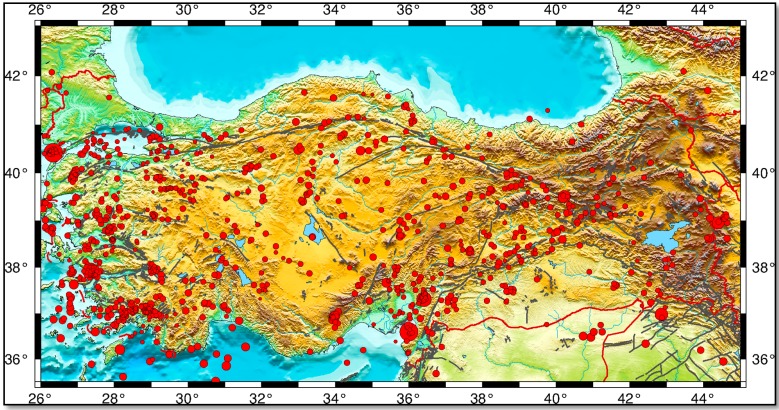
Seismicity of Turkey and surroundings. Red circles mark earthquakes. The circle size is proportional to the magnitude of the earthquakes (magnitude equal to and larger than 4.0) [7]. Bold black lines indicate the faults [8]. The figure is generated by the Generic Mapping Tools (GMT) package of Wessel and Smith [9].

Turkey is one of these male-dominant societies and is a country that experiences a high rate of natural disasters. The distribution of significant disasters in Turkey (Figure 1) shows that risks due to earthquakes are high (61%) [5], indicating that Turkey is an earthquake-prone country. Based on the available earthquake catalogue information [6], 1175 destructive historical earthquakes were identified in this area between 2100 B.C. and 1900 A.D. In addition, the catalogue information [7] indicates (Figure 2; faults from [8] and generated by the Generic Mapping Tools [9]) that in the instrumental period (after 1900), 149 destructive earthquakes occurred before the Gölcük-Kocaeli earthquake (17 August 1999, moment magnitude Mw = 7.6) and 97,203 people lost their lives because 578,544 structures were destroyed or severely damaged. According to information from the Ministry of Public Works and Settlement General Directorate of Natural Disasters, in the Gölcük-Kocaeli earthquake, 66,411 houses and 10,901 workplaces were severely damaged, 67,242 houses and 9927 work places were moderately damaged, 80,160 houses and 9712 work places were slightly damaged, and 17,479 people lost their lives [10,11].

When the numbers of demolished and severely damaged buildings and the loss of life from the Gölcük-Kocaeli earthquake are compared with the data obtained for the period immediately prior to the earthquake, the numbers show that 14% of buildings were demolished and severely damaged by the earthquake, and 18% of the population lost their lives. These figures are only for one earthquake, indicating the significance of the risk. The total number of people who were affected at different levels by the Gölcük-Kocaeli earthquake is roughly 20 million [12], indicating that this was an urban earthquake [13]. The Gölcük-Kocaeli and other urban earthquakes have provided insight into the level of risk experienced by women and the resources that they provide in such emergencies. Therefore, this earthquake has motivated gender-based scientific evaluations of earthquakes in Turkey.

According to the calculations, there is a 62% probability [14] that a destructive earthquake of at least M = 7.0 will occur in the Marmara Region. This probability highlights the urgency of reducing the risks to women, who have significant roles in pre-earthquake and post-earthquake rehabilitation/reconstruction, and of utilizing women as resources. In particular, new disaster management strategies with a gender approach are required because a destructive earthquake is likely to occur in Marmara in the near future. Gender issues occur in all geographic sites and have occurred for hundreds of years; however, they differ depending on the region and culture. In association with disasters, these issues may lead to increased losses, as observed in nearly every disaster worldwide. However, awareness of gender issues has not been raised during pre-disaster phases or during loss-reduction and preparation processes.

The concept of gender refers to the forms, roles, opportunities and relationships that are constructed socially for men and women [15]. Thus, gender defines the cultural, social, religious and historical characteristics of men and women as well as the roles and responsibilities assigned to men and women by society and social expectations. Specifically, gender defines the roles of women as mothers and men as fathers, enforces work roles, and dictates the responsibilities for child care and earning a living for the family. These roles and responsibilities have been assigned to men and women in different cultures throughout history and in different geographic areas. These facts are even more complicated when factors such as age, marital status, social class, ethnicity, religion, and refugee status interact [15,16]. Gender roles refer to the roles and responsibilities expected from women and men based on gender differences. In this context, femininity and masculinity define the gender roles that society deems suitable for women and men. Gender equality refers to being free from discrimination when taking advantage of opportunities, allocating and using resources and accessing services based on one’s sex [16].

The concepts of gender and gender equality originate in feminist theory [17]. Feminist theoreticians have produced policies to improve women’s social status through observations of the unequal status of women in society with the goal of ultimately creating a society that includes gender equality. These theoreticians criticize the structure of social life as well as traditional and social theories that consider this structure normal. Social theories constructed from a male perspective remain insensitive to gender-based problems and ignore women’s experiences and skills.

Until recently, gender relations and dimensions of gender equality have been completely overlooked in disasters, and discussions of disaster management that include gender are limited. These discussions began with Fothergill [18], who explained that there is “…a pattern of gender differentiation throughout the disaster process” and “…the differences are largely attributed to childcare responsibilities, poverty, social networks, traditional roles, discrimination, and other issues of gender stratification”. This approach was the first to associate “gender” with disaster management. However, as Enarson *et al.* [19] stated, the gendered dimensions of disasters remain underreported and poorly managed. To address this issue, The Platform for Action Fourth World Conference on Women (Beijing, 1995) recognized the effects of natural disasters on women, and similar efforts must be increased. In 2000, the Beijing Platform for Action stated that the social and economic effects of natural disasters and epidemics are not made visible as an enforceable policy. The Preparedness Against Disasters and Emergencies project has been established in conjunction with the Health and Social Security Center (SSGM), related ministries and institutions to maintain effective preparation activities and reduce the adverse effects of disasters on women and children (2001–2005 Turkish Republic-UNICEF cooperation program). Studies on other affected groups continue within these institutions.

Increases in the number of disasters and associated damages were discussed at the World Conference on Women, 23rd Special Session, entitled Women 2000: Gender Equality, Development and Peace for the Twenty-First Century. One consensus of this meeting indicates that current disaster management approaches do not address disaster prevention and mitigation from a woman’s perspective and provide insufficient responses, increasing disaster losses. As a result, initiatives for policies that are responsive to gender have begun to be implemented. At the Yokohama World Conference on Natural Disaster Reduction held in 1994, the role of social sciences in research and policy production and settlement was emphasized, and the relationship between disaster damage reduction and sustainable development was underlined in the interim report of the Tenth Year of International Natural Disaster Reduction. Furthermore, the need to overcome society’s limitations with regard to natural disasters as well as the importance of strengthening women in each phase of disaster management were emphasized.

In Turkey, the subject of gender in relation to disasters was addressed in an interdisciplinary (e.g., academicians, public officials, voluntary agency members, emergency and medical technicians) workshop, the 3rd International Workshop on Gender and Disaster, which was held by Kocaeli University, Faculty of Economics and Administrative Sciences in October 2008 with the support of the Office of U.S. Foreign Disaster Assistance (OFDA). After that, Strategy C.2.3 A special arrangement for individual groups at risk was included in the National Earthquake Strategy and Action Plan, 2012–2023, a report published in February 2012 by the Disaster and Emergency Management Presidency (AFAD) [20]. This document states:

Studies in which individual consideration is provided to each group that forms a great majority of the society, including women, children, old persons and disabled persons, should be conducted and the results of these studies should be included within the disaster management system. Increasing the ability to cope with disasters and decreasing vulnerability are functions of social ties, power relations, knowledge and abilities, gender roles, health and economic development levels and settlements of persons and social groups. In this process, the weakened position of women, children, old persons and disabled persons should be included as a factor that increases their vulnerability.

This strategy is consistent with the approach advocated here. However, it cannot ensure that women are no longer at risk or that they will be used as resources in disasters. The sharing of first-hand experience, observations and records by men, women, transgender groups and Non-Governmental Organizations (NGOs) was instructive in understanding the problems of disaster management and developing solutions. However, although disasters occur frequently in Turkey, the site data could not be evaluated based on first-hand experiences, observations and records because such information was insufficient. This event motivated us to document site observations and interviews with local people who experienced the earthquake to generate gender awareness of the disaster.

Purposes in this study are to determine how to eliminate the risks to women in all phases of a disaster and to contribute to the evaluation of women as resources. Studies on the role of women in disasters are insufficient. Therefore, this subject is evaluated in this study using examples from Turkey that may be generalized globally to contribute to improvements in the reliability of current management practices and to highlight the role of women in disaster management (specifically earthquakes). Presenting gender-based requirements and inequalities in disaster events and improving overall gender awareness of disasters—including why women must be active in all phases of a disaster, especially in disaster management—are the main goals of this article. 

## 2. Approaches and Observations

As it is pointed out earlier, disaster management is a multi-disciplinary task with the various phases of the disaster management cycle (mitigation, preparedness, response and rehabilitation). Briefly, these terms refer to [21];

(1) Mitigation: aims to minimize the results from a disaster;
(2) Preparedness: planning how to respond;
(3) Response: initial actions taken, as the event occurs to minimize the hazards created by a disaster;
(4) Rehabilitation: the stage returning the community to normal.

**Figure 3:**
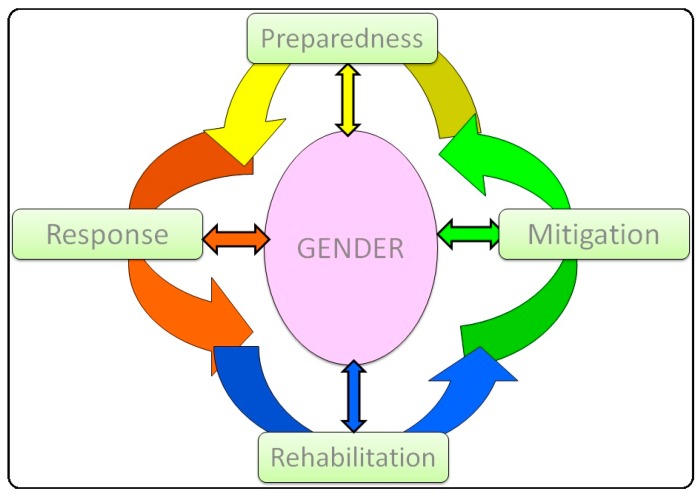
Schematic representation of a gender-oriented disaster management cycle.

Due to the lack of data on gender specific issues in disasters, it cannot be applied a quantitative analysis of the problem that focus on. Therefore, perspective in this study includes how an effective disaster management plan should be constructed; in order to reduce the negative impacts of disasters caused by gender inequality, and improve disaster responsiveness. It is offered a conceptual foundation for gender based disaster management cycle (Figure 3). For this purpose, first it is pointed out the different needs, and priorities of women and men following disaster from field observations, then it will be introduced the risk status of women and possible solutions for related issues are presented for every stage of disaster management cycle.

### 2.1. Requirements and Inequalities in Disaster Priorities

Women and men are affected differently by disasters because of their physiological differences, physical and psychological vulnerabilities, social perceptions of their roles and responsibilities (mother *vs.* father), and unequal access to and use of economic resources and education facilities, which necessitates specific actions and priorities in a disaster event. The related genetic, psychological, physiological, legal, sociological and educational differences are summarized in Table 1. Determining the socio-anthropological characteristics of men and women can help in evaluating differences in relation to disaster management. Moreover, emergency action plans for disaster management that include maps of country-wide social profiles should be considered.

**Table 1:** Different needs and priorities of men and women following disasters in Turkey. Genetic and psychological differences are discussed together.

Differences	Women	Men
Genetic Psychological	Raising children Children’s safety Appropriation Psychological resistance	Considers himself head of household and holds decision-making power Loses the power of management because of the disaster and loses the leadership role Loses leadership and decision-maker characteristics Becomes aggressive, leading to domestic violence and family disruption Abandons parental responsibilities Psychological trauma
Physiological	Less physical power Women are passive, and the use of their power occurs through long processes	More physical strength Trauma because of failure to fulfill responsibilities and loss of power in a short time
Legal	Lack of legal equality between men and women	Predominance of laws in favor of men
Sociological	Woman’s family Mother Women (both individually and throughout society) are disadvantaged because of their lack of legal rights	Head of household Father Men (both individually and throughout society) hold decision-making power with legal support
Education	Poor educational facilities Educating women and girls on disaster plans remains secondary	Men are prioritized educationally

The differences between men and women may vary by country, city and type of area (rural *vs.* urban). In male-dominant societies with rules that are structured by tradition, women often cannot travel outside the household without receiving permission from the head of the household, and they cannot transition from their own private areas to social life. Therefore, women are socially forced to stay at home with their children even during destructive disasters, which exposes them to additional risks. These conditions are similar to those observed in Turkey and many places around the world. For instance, in the Van earthquake of 2011 (Mw = 7.2), which occurred in southeastern Turkey, Bediha Özgökçe Ertan, a volunteer at the Van Bar Association Women’s Rights Solidarity Center, observed that “during the earthquake, which occurred on Sunday at daylight, the number of women and children among the ones who died was higher because women and children were at home” [22]. Similarly, in the cyclones and floods that occurred in Bangladesh in 1991, the death rate among women was five times higher than that for men because women did not leave their homes due to tradition that dictates that it is unacceptable for women to go out alone [16]. Such situations clearly highlight the importance of gender awareness in disaster management.

### 2.2. Gender Awareness in Disaster Management

Disasters are events that reveal the striking degree of gender inequality (injustice). The significant contributions made by women during disasters despite men’s quantitative and cultural dominance require the development of gender-focused disaster management. Due to their traditional roles in conservative societies, women may be more resilient, and multi-tasking individuals (e.g., adopting the roles of mother and spouse simultaneously) may provide resources during disasters. Therefore, gender’s place at the center of the disaster management cycle (Figure 3) reflects a consideration of women as resources in all phases of disaster management, which is shortly explained in Chapter 2. The implementation of gender equality policies must not be neglected in disaster management policies, plans and strategies.

### 2.3. Why Have Women Become An Important Factor in Disaster Management? Are Women both at Risk and Important Resources in Disaster Management?

An examination of disasters based on gender from the perspective of a disaster manager shows that men also suffer as a result of society’s expectations. However, women are the focus of this study because women’s gender-related problems far exceed those of men. Here, girls and boys are included in the terms “women” and “men”, respectively.

The role of women in disasters was recognized with the emergence of urban earthquakes. In Turkey, the role of women in rehabilitation was clearly observed when 20,000 lives were lost and approximately 100,000 houses became uninhabitable after the Gölcük earthquake [23]. Although women make significant contributions to earthquake relief, their usefulness as resources in disasters is limited because they cannot participate sufficiently in plans and scenarios because of their gender.

Women are at the greatest risk of incurring damage in disasters; although they have the ability to provide valuable resources in all phases of disaster management, their lack of qualifications limits their usefulness. This disparity allows us to address the subject of disaster management from two different perspectives: risk and resource. In particular, after the Gölcük earthquake, women suffered because of their gender while also providing important help in the aftermath.

### 2.4. Why Are Women included in the Risk Group? In which Situations Do They Become a Risk?

Globally, the male perspective is dominant, although this situation has diminished somewhat in developed countries. In under-developed or developing countries, however, gender equality may be ignored in practice due to the social structure and tradition even though gender equality is ensured by law. Therefore, women may have limited opportunities for education, and it may be more difficult for women to access resources. Additionally, women do not participate in decision-making processes. This inequality is further promoted by the roles appointed to women by societies, traditional behavioral patterns and relationships.

Disabled and elderly persons, children, women and people with chronic diseases (e.g., diabetics, dialysis patients) are included within the high-risk group. Risks for women must be determined individually with regard to communication, security, accommodations, water sanitation, health, nutrition, psychological support and transportation, and such considerations must be included in emergency action plans for the stages of the disaster management cycle (Figure 3).

An examination of disasters from a gender perspective shows that women suffer more because of their roles. For example, four times more women than men died in the devastating tsunami that occurred after the earthquake in Asia (26 December 2004) because of women’s gender roles and traditions at this society: many women returned to save their children, did not know how to swim, or could not climb trees, and their long skirts frequently prevented them from escaping [24]. In disasters, women suffer from sexual harassment and violence and experience numerous difficulties, such as nutritional and hygienic difficulties during pregnancy and breast-feeding.

The violence and pressure toward women that already occur in male-dominant societies escalate even more during disasters, leading to psychological trauma and increased risks to women during disasters. Such issues must be regulated by law, and necessary precautions must be taken to ensure that law enforcement groups protect the rights and security of each individual regardless of gender.

### 2.5. Why Are Women an Important Resource in Disaster Management?

Recognition of the way that women’s genetic and physiological differences/features and skills contribute to disaster relief has begun to emerge after recent large-scale disasters and urban earthquakes. These can be briefly summarized in the following categories:

- Resilience and ability to adapt to the rehabilitation period of disaster management
- Perception of risk
- Versatility and ability to observe and evaluate details
- Ability to resist psychological trauma
- Natural ability to provide group therapy
- Resourcefulness in search and rescue
- Performance in NGOs

According to post-disaster field observations in this study, duties such as cooking, childcare, and cleaning that are attributed to women based on their traditional gender roles are assumed by women because they have become reflexes. Women’s ability to focus on more than one job and to perform multiple tasks simultaneously provides them with rapid problem-solving skills in disaster environments. This versatility imbues women with the ability to persevere and provides them with additional strength in disaster events.

Men may have difficulties recovering from the shock of great trauma (such as a disaster) if they are unaccustomed to expressing emotions. Men who are unable to talk, cry or share because of their assigned gender roles, which include restrictions on crying and talking and requirements to be serious, may behave as though they do not care about small problems. Furthermore, women may perform better under the pressure of disaster situations because they have often developed a psychological immunity to the problems that they manage in their daily lives. This characteristic may provide women with more strength than is observed in men during disasters. Women resist succumbing to depression because they are accustomed to managing multiple problems in their daily routines. Disaster recovery is a long process, and women adapt more quickly to post-disaster duties and use their strengths more efficiently.

The characteristics that women have developed spontaneously to overcome problems arising from gender differences have enabled them to naturally improve their organizational and coordination skills. When the development of these skills is supported by education, the services provided by women can be more efficient during disaster management, and women may become valuable resources and participants in organizing and coordinating disaster management.

NGOs have played important roles in disaster management in Turkey, especially in the Gölcük earthquake of 1999. These organizations reached the disaster area in time to deliver aid because they moved quickly. However, their assistance only lasted for a short time, and their participation in the rehabilitation process was short-lived as well. In the Gölcük earthquake, the search and rescue teams rescued approximately 1000 people. In contrast, approximately 10,000 people were rescued by their neighbors. As a result, neighborhood disaster volunteer (Mahalle Afet Gönüllüleri, MAG) NGOs were established to organize inhabitants for search, rescue and rehabilitation.

The subject of women in search and rescue must be addressed from two perspectives: women participating in search and rescue teams at the site and women participating as disaster victims. In recent years, only men have participated in search and rescue teams in many countries because of their physical strength. Today, the successful inclusion of women in search and rescue teams is increasing. For instance, no women participated in the search and rescue operations after the Gölcük-Kocaeli earthquake. In the following years, women began to participate on these teams more frequently, with a 70% participation rate by women in the neighborhood disaster volunteers (MAG). These women should be cited to act as role models for other women in any outreach activities related to search and rescue after a disaster, helping to understand the new role that women should play to bring ethics in a modern and risk society [25].

Researchers and rescuers noted that under the wreckage, they encountered and were affected by mothers who died hugging their children. In disasters, mothers continue to fulfill their roles as protectors, even at the risk of death. In the Van earthquake, women who had babies continued protecting and breast-feeding their babies even during the disaster evacuation activities.

The contributions of NGOs are of great importance in disaster management. In Turkey, although women’s resourcefulness has not reached the levels desired by NGOs, a remarkable increase has been observed in the number of women participating in NGOs since the urban earthquakes of 1999. This increase has mostly occurred in organizations that work to prevent disasters and was caused by a disruption to traditional male-female limitations and a recognition of the necessity of women during earthquakes. These changes have allowed women to serve as resources.

**Figure 4:**
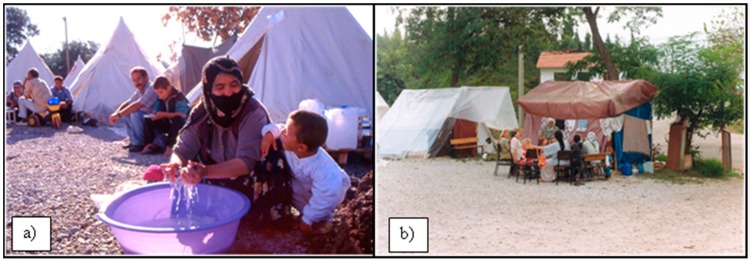
Women continue their struggle for survival after earthquakes; (**a**) a woman with children in a tent city in the Van earthquake [26]; (**b**) women’s compliance during post-disaster rehabilitation after the earthquakes in Gölcük-Kocaeli and Adapazarı [12].

In past disasters, women have played important roles in environmental and educational activities, pre-disaster preparation programs, environment management and disaster loss reduction while also protecting their own homes. In addition, traditional women’s gender roles allow them to perform tasks such as cooking and laundry in groups. This perpetuates their normal lives after disaster and creates natural group therapy through talking and sharing, which reduces the effects of the resulting psychological trauma (Figure 4a,b). Because of their behavioral characteristics, women may overcome disaster-based traumas sooner and may provide active roles for longer periods during disaster management processes, thus enhancing their resource characteristics.

### 2.6. The Gender Factor with Minimum Standards in Disaster Management

Based on observational data from disasters, it is important to apply international standards (Humanitarian Charter and Minimum Standards in Disaster Response), especially in the response and rehabilitation phases. Gender must be effectively factored into these standards. Previous disasters have shown that certain areas, especially women’s security, health, hygiene, accommodations, and nutrition for breast-feeding and pregnant women and women with children, contribute to incremental increases in women’s suffering even after surviving disasters. It is clear that women’s suffering can be reduced if certain standards are established that consider gender. These standards can be provided with the help of gender maps.

### 2.7. The Role of Education in Overcoming Gender Problems in Disasters

Disaster plans have not been constructed from a gender perspective. Therefore, disaster managers must be provided with accredited training, and extensive sustainable education is required, from compulsory to higher education. An educated society can reduce losses during disaster management. The risks encountered by women because of a lack of education will decrease through proper education, thus eliminating these risks and enabling women to serve as resources. For example, a woman who is provided with training in first aid or fire-fighting can be a resource in all phases of disaster management.

## 3. Results and Discussion

The risk status of women and solutions for issues related to security, accommodations, health and nutrition are presented in Table 2 for the stages of disaster management cycle (Figure 3). According to the health module, which is included in the minimum disaster relief standards in the United Nations humanitarian aid agreement, women can exercise their health care rights in three areas: (1) reproductive health and family planning, (2) psychological and physical health, and (3) access to health services. Although this state of affairs is the case under normal conditions, unfavorable conditions for women’s health increase incrementally during disasters and lead to greater risks for mothers, nursing mothers and pregnant women, which is similar to the situation in developing and underdeveloped countries. These risks should be reduced and converted into resources in national disaster master plans and in regional emergency action plans devised during the loss-reduction and preparation periods before disasters.

**Table 2:** Modules that should be included in emergency action plans: risk status of women during disasters and solutions during different phases of the disaster management cycle.

Module	Risk Status of Women	Solution				
		Before Disaster			After Disaster	
		Mitigation	Preparation		Response	Rehabilitation
Safety and Security	Abuse, rape, extortion, risk of injury and death because of carrying precious jewelry, risk of the trafficking of women	Security planning and training (staff and residents)	A manual describing women’s legal rights and informational trainings and plans prepared for scenario exercises and logistics		Disaster area safety and security, security assistance with search and rescue teams	Tent site safety and security
Housing	Design of tent areas without regard for women’s mental and physical vulnerabilities	Attention to planning, meeting minimum standards but also being gender sensitive	Gender-sensitive logistical support		Number of women at the evacuation area, safe shelter and field records	Gender-sensitive tent sites, toilets for women and children nearby, playgrounds for children, proper venues for breast feeding
Health	Risk of miscarriage and premature birth, aggravated gynecological complaints	These special cases should be considered in master plans and in planning and public health policy; gynecologists and pediatricians should have input		Medical supplies and logistical preparation based on women’s health, such as diapers, sanitary napkins	Preparation of women-sensitive field hospitals, provision of female gynecologists, doctors and psychiatrists (when necessary)	Health screenings for women, periodic inspections and drug support, and detailed medical record keeping
Food	If pregnant women’s special food requirements are not met, mother and baby may be in danger; the situation may be life-threatening if breast-feeding mothers cannot meet their special food needs	Planning should comply with women’s specific periods and conditions		Proper food preparation for women, babies, and children	Food support prioritized for pregnant and breast-feeding women, infants and children	Sustainable food-related programs

The needs of the groups that are most affected by disasters must be considered and included in disaster preparation [16,21]. When violence against women, such as injuries, robberies (e.g., for jewelry), murder, divorce and post-divorce economic problems that can occur during disasters are evaluated based on women’s legal problems, it becomes clear that regulations for women are required. 

In general, perceptions of earthquakes vary according to regional and cultural differences. Regardless of these differences, cooperation begins immediately after a disaster, revealing the altruistic characteristics of society. To reduce women’s risks and use them as resources, it is necessary to reveal of these differences in detail through scientific studies.

NGOs such as bar associations and the Contemporary Lawyers Association work effectively to teach women their legal rights in relation to disasters and to help them obtain their rights. In this context, training should be provided for women, and guides should be prepared and distributed during the disaster preparation phase before a disaster strikes.

The actors who have a role in disaster management [4,24] are listed below in terms of their authority and responsibilities:

- Political authorities and institutions that govern the state
- Local authorities
- Security forces
- Mass media
- Universities
- NGOs
- Professional chambers
- Earthquake specialists
- International institutions
- The public

Disaster management training for women should vary in relation to these actors and where their help will be required. Supporting efforts to create gender awareness among all actors in the disaster management process should be a priority for all disaster managers.

## 4. Conclusions

In many parts of the world, natural, technological and human-driven disasters have taught us that a design that is responsive to gender is required for successful disaster management, particularly when creating disaster management plans and strategies. Worldwide, gender-focused studies are not sufficient, which increases the need for gender awareness. Disaster management policies and strategies will be even more effective at reducing loss of life and damages with the implementation of gender policies. Globally, response and rehabilitation occur after disasters. The urban earthquakes in Turkey showed that reducing loss and preparing for disasters are of great importance. Therefore, new disaster management plans must be structured to eliminate the risks to women and to utilize women as resources. Policies on gender and disaster management must be considered and applied together.

One of the keys to a successful disaster management plan is to correctly analyze social patterns, which can be accomplished through socio-anthropological studies. Nationwide social profiles must be created, mapped (gender maps) and considered in emergency action plans. Another key to successfully minimizing disaster losses that result from gender problems is to focus on gender-based issues by evaluating sociology, law and psychology along with sciences such as engineering and medicine. A gendered perspective on possible disasters must be indicated on maps (women’s risk and resource maps), and an information system network must be created that considers risks and resources from the smallest regions to general areas.

In disasters, logistics and health aids should be responsive to gender. Adequate infrastructure and appropriate specialists should be made available for women based on differences in needs and priorities. During pre-disaster loss reduction and preparation, countries should prepare disaster health master plans that consider gender-based risks and reduce these risks by using regional emergency action plans and shifting women’s role to that of a resource. In each phase of disaster management, international disaster aid standards should be applied in gender-responsive plans and applications.

In addition, women should be provided with access to education opportunities and should play a sufficiently active role in planning, risk prevention, decision-making, search and rescue and rehabilitation throughout the disaster management cycle. It is of vital importance to build a structure that does not de-prioritize or trivialize female site keepers and their experiences. This perspective should not be overlooked in analyses, plans, decisions or evaluations because the inclusion of this perspective is the only method of achieving gender justice. Gender-oriented disaster training should be provided within the scope of general education (especially for male managers and heads of households).

Reducing risks for women and converting women to resources is a debt required of developed societies to other societies. It is also important in terms of creating a contemporary work policy that requires international institutions and NGOs to use their civil initiatives to reduce risks and improve the roles of women while accelerating the creation of common disaster policies in regulating international policies. Only in this way can a fair, ethic, balanced and decent world order be assured in the fight against disasters. It must not be forgotten that the greatest disaster is underdevelopment.
